# Qualities, subjectivity and spatial extension: is there a common factor?

**DOI:** 10.3389/fpsyg.2026.1813385

**Published:** 2026-06-29

**Authors:** Bruno Forti

**Affiliations:** Department of Mental Health, Azienda ULSS 1 Dolomiti, Belluno, Italy

**Keywords:** extended information theory, feature qualities, phenomenal analysis, phenomenal consciousness, spatial extension, thingness

## Abstract

According to the Extended Information Theory, conscious vision allows us to know how objects extend into the space they belong to. In this way, consciousness compensates for some of the limitations of Neuro-Computational systems, which handle Point-Like Information. The aim of this paper is to explore the potential links between spatial extension and two other properties of phenomenal experience: subjectivity and the qualitative aspect. One way to investigate these aspects and develop a coherent model for explaining subjectivity, qualities and spatial extension is to identify a property that underlies them. I have identified this property in an aspect of appearance: thingness. Thingness consists in the materialization of something that extends into space. The other properties are derived from it. Through spatial extension, we gain immediate knowledge of how an object is made. Subjectivity implies a structure in which a third-person world forms within a first-person world. Consequently, egocentric knowledge is formed that encompasses the subject itself. With quality, we know how something materializes based on its degree of differentiation from other qualities. The way in which it materializes in feature qualities can explain both their relational and categorical phenomenal character. The knowledge conveyed by qualities transforms existing unconscious knowledge into conscious knowledge. It is an accessory function of consciousness and is linked to the extended nature of objectual contents. I propose a hypothesis about how these qualities may originate from physical processes in the brain.

## Introduction

The Extended Information Theory (EIT) (Forti, 2025) jointly addresses issues related to the nature of consciousness and those related to its functional role. It is based on the property whereby all conscious content extends in some way into space. Conscious vision allows us to know how objects extend into the space they belong to and, at a more complex level, the structural aspects of the physical world that surrounds us. Extended Information is characterized by the fact that its informational units are objects or entities that are spatially extended in some way and cannot be reduced to point-like entities.

The argument that consciousness handles Extended Information acquires an important meaning if we note that the information processed by a Non-Conscious (NC) system is necessarily point-like information. Such a system has limitations in handling information related to a world made up of spatially extended objects. The term Non-Conscious process refers to a process that occurs in the absence of consciousness. To a certain degree of approximation, the acronym can also be interpreted as Neuro-Computational and include both artificial processes and processes carried out by the brain in the absence of consciousness (Forti, 2009).

I introduce the concepts of thingness and spatial extension, which represent a redefinition of concepts that I discussed and explained extensively in the previous article (Forti, 2025). Thingness, that is, the materialization of something that extends into space, corresponds to the fundamental property of consciousness whereby its objectual contents extend into space. Spatial extension is the term attributed to the property whereby conscious contents extend in some way in space. In the first case, the emphasis is placed on the objectual and extended nature of conscious contents, in the second on their mode of extension in space. These concepts have been redefined for the sake of conciseness and to adapt them to the new context.

In this paper, I explore the potential links between spatial extension and two other properties of phenomenal experience: subjectivity and the qualitative aspect. The scope of this paper is limited. By limiting myself to perceptual consciousness, I focus on specific aspects of qualities and subjectivity. In the first case, I focus solely on feature qualities, excluding an important domain such as that of feelings and emotions. In the second case, I refer exclusively to the knowledge of the spatial extension of the subject’s body. I do not address the question of the subject as an agent and as a motivational entity.

Within the limitations described above, this paper aims to highlight a common factor underlying the properties of experience. A common factor lies at the root of diverse phenomena such as the spatial extension of the contents of consciousness, subjectivity, and feature qualities: thingness, which is understood as the phenomenal materialization of something that extends into space.

In light of this common element, I examine the possible functional aspects of subjectivity and qualities. The question of the functional role of consciousness remains unresolved and, at the same time, is underestimated. Theories of consciousness proposed in recent decades “are concerned primarily with how consciousness arises, and only secondarily, if at all, with the biological function of consciousness” (Earl, 2014). The conscious structure makes it possible to distinguish between first- and third-person perception. In other words, it allows us to clarify how knowledge of the body’s spatial extension – being subjective – differs from knowledge of the spatial extension of a visual object. Feature qualities possess an extended nature, too; they overlap with the object and constitute an extended form of knowledge that is related to that of the object. As a result, we will know how a certain quality extends in relation to the object’s extension. Furthermore, feature qualities transfer existing unconscious knowledge to the level of extended conscious knowledge. Thingness, being an expression of the relationship between the object and the surrounding space, is a subpersonal property and is meant to be describable in the third person. Consequently, subjectivity and qualities, deriving from this common factor, can also be conceived in the third person.

## Methodological aspects

I will limit myself to analyzing qualities understood as feature qualities. I make a simple distinction that is not often made in the literature on the topic (Prinz, 2004, 2012; Kriegel, 2009), even though it is part of the meaning of quality in common sense. Quality is (1) a characteristic or feature that someone or something has: something that can be noticed as a part of a person or thing; (2) how good or bad something is (Encyclopædia Britannica, 2023). The first meaning has its own autonomy. This is because it has to do with knowledge that must be distinguished from the pleasure or displeasure that may accompany it.

I will also limit myself to analyzing subjectivity in its simplest aspects, relating to bodily perception, excluding those related to consciousness as agency. The latter have to do with the subject as an agent, with emotions, and with the hedonic value of qualities (LeDoux, 1996; Damasio and Carvalho, 2013; LeDoux and Brown, 2017; Damasio, 2021; Solms, 2021; Engelen and Mennella, 2023; Frumento and Menicucci, 2024). On the one hand, this is a limitation in my approach. On the other hand, investigating consciousness by considering it as a whole that encompasses all its possible articulations (Baars, 1997; Rosenthal, 1997; Crick and Koch, 1998; Dehaene and Naccache, 2001; Tononi, 2008; Dehaene, 2014; Hameroff and Penrose, 2014; Tononi and Koch, 2015; Brown et al., 2019; McFadden, 2020, 2023; Seth, 2021; Hunt et al., 2024; Zhi and Xiu, 2023; Strupp, 2024) may be a more significant problem.

I have called the method I adopt in this paper phenomenal analysis (Forti, 2024a). Quite simply, its objective is to identify the fundamental structure of consciousness by analyzing very simple forms of experience. One of the difficulties in understanding consciousness lies in the belief—which I view as erroneous—that its simplest aspects are non-structural in nature (Loorits, 2014). The phenomenal analysis of the simplest aspects of experience allows us to identify the structure of consciousness within consciousness itself.

Sensations such as the redness of red or the painfulness of pain are inseparable from the context of the experience to which they belong, making qualia appear as phenomenal artifacts. Hence, the simplest forms of experience are not found in sensations, but rather in forms of perception in which the qualitative aspects of consciousness necessarily have relational significance (Forti, 2024a). Furthermore, this analysis leads us to hypothesize that the structure of an early visual experience is constituted by a Hierarchy of Spatial Belongings (HSB) nested within each other. Every spatial belonging is made up of a primary content and a primary space, which is not perceptible. In this sense, the structure of consciousness is counterintuitive because it is also made up of hidden parts. Box 1 lists what phenomenal analysis can detect (Forti, 2024b).

BOX 1 **Third-person properties that phenomenal analysis allow us to identify**

- Even in its simplest forms, experience is made up of relationships between qualitatively connoted parts. Qualitative components of conscious field are interdependent and form a unitary whole.

- The unitary set of qualities found in early vision—such as those related to being an object, background, or detail—constitutes the explanandum of the simplest forms of consciousness.

- The fundamental relationship of visual consciousness is between content and surrounding space. This relationship manifests not only between objects and backgrounds, but also in counterintuitive ways. Consciousness is not just what appears. Its organization is based on a HSB. Each HSB is formed by a primary content and a primary space that is not perceptible in itself.

- Consciousness involves a high degree of overlap between Spatial Belongings, which is possible thanks to the phenomenally negative nature of primary spaces.

- Its contents extend in a certain way into the space of belonging. This property enables an extended knowledge of images relating to the world that is current, spatially structured, image-based, private, and integrated.

- Consciousness behaves like a self-organizing extended reference system, in which conscious knowledge seems to be based on the degree of deviation from a condition of equilibrium of the object in its relations to the space to which it belongs.

My analysis is derived from the Gestalt approach, some of whose assumptions I discussed in a previous paper (Forti, 2015). Gestalt laws require the presence of multiple contents in relation to each other in the field. In a simple context such as a figure on a homogeneous background, the Gestalt approach focuses exclusively on the figure-background relationship. If instead the key element of perception is defined as the relationship between content and surrounding space (Box 1), the fundamental function of consciousness can already be identified at this stage. In any case, phenomenal analysis shares with Gestalt theory the distinction of essentially being a phenomenological approach (Merleau-Ponty, 1945; Kanizsa, 1980, 1991). Gestalt theory addresses simplified perceptual situations. My approach addresses even simpler situations, characterizing itself as a kind of elementary phenomenology (Husserl, 1900–1901/2001; Zahavi, 2018).

Although phenomenology focuses on structural aspects of consciousness, its aim is to identify, through first-person observation, structures that are also, in a sense, first-person. The goals of phenomenology do not involve the search for an explanation of consciousness, as phenomenology addresses phenomena as they manifest themselves in the intentional consciousness of the subject (Gallagher and Zahavi, 2008). However, if the goal is a scientific theorization of consciousness, a first-person event should be described—and possibly explained—from a third-person perspective (Drayson, 2015). Third-person structures can be more easily investigated empirically in terms of their possible biological and physical assumptions (Nagel, 1974; Searle, 1992; Chalmers, 1996).

To summarize, the classical approach to phenomenal consciousness tends to underestimate—if not entirely overlook—the structural aspect. On the other hand, the structures of consciousness highlighted by the phenomenological approach are invariably first-person. My analysis also uses observations that are necessarily first-person. However, an analysis focused on elementary aspects of visual experience can highlight a structure that can be described in the third person, in which what happens does not necessarily depend on the relationship with a subject. Such structure is based on a relationship between objectual content and surrounding space (Box 1). The relationship with space is obvious in the case of the background, but it is also present in a detail, in qualities and even in the darkness we perceive with our eyes closed (Forti, 2024a).

This is not as counterintuitive as it might seem. Gestalt laws are based on relationships between elements of the visual field that have little or nothing to do with the subject. When dealing with more strictly subjective experiences, such as bodily ones, identifying the third-person structures I have identified in vision seems difficult. I will explore if it is instead possible to somehow overcome the seemingly unbridgeable gap between first- and third-person ontologies (Searle, 2004).

## The fundamental property of consciousness

For most scholars, being conscious implies having a phenomenal experience (Chalmers, 1996; Block, 1995; Zahavi, 2005; Horgan and Kriegel, 2007; Tononi and Koch, 2015; Feinberg and Mallatt, 2020; Cleeremans and Tallon-Baudry, 2022; Newen and Montemayor, 2025). Consciousness is closely linked to the concept of phenomenon and its etymological meaning of appearing (O’Shaughnessy, 2000; Yolton, 2000; Krioukov, 2020). What is not obvious is how we should understand appearance and phenomenon, terms which I use interchangeably.

The Kantian idea that phenomena are things as they appear to us is well known (Kant, 1781–1787/1998). In Husserl’s phenomenology, a “phenomenon” is the object or structure of experience as it is perceived and experienced from a first-person perspective, rather than an as objective, material reality (Husserl, 1913/1982). According to Nagel (1974), a being is conscious only if there is “something that it is like” to be that creature, that is, some subjective way the world seems or appears from the creature’s mental or experiential point of view (Van Gulick, 2022). However, these conceptions of the phenomenon refer to a construct that presupposes both the way things appear to us and a relationship between subject and object. The problem does not only lie in the way the world appears to us. It also lies in the mere fact of appearing (Revonsuo, 2006; Whiting, 2016; Merlo, 2020). Moreover, the subject is not just something *a priori* (Husserl, 1913/1982; Zahavi, 2018). The subject is part of appearance, albeit differently from the object (Heidegger, 1927/1962, Merleau-Ponty, 1945; Legrand, 2007; Gallagher and Zahavi, 2008; Glasgow, 2018; Gallagher, 2023). A definition must be established beforehand to justify the subject and its relationship with the object.

Thus, we should break the phenomenon down into three components: something that appears, the way in which it appears, and a subject to whom the appearance is directed. The first represents the fundamental property of consciousness. We have seen that the fundamental structure of visual consciousness is grounded in the relation between object and space. This relation occurs between an inner region that has the properties of an object, of a material thing, and an outer region that has the properties of a space in which the object is extended. The fundamental property of perceptual consciousness derives from this relation.

In fact, at least in a relative sense, appearance is something that is not evenly distributed throughout the conscious field, but it concerns some regions of the conscious field to a greater extent than others. Moreover, the latter seem somehow necessary for perception to occur. “A figure on a background … is the very definition of the phenomenon of perception, that without which a phenomenon cannot be said to be perception at all. The perceptual ‘something’ is always in the middle of something else, it always forms part of a ‘field’” (Merleau-Ponty, 1945). From this point of view, one could say that, since the relationship between object and background involves the existence of contrast, the conditions for the emergence of an elementary form of phenomenal experience do not depend on the – metaphorical – coming on of the light of consciousness, but on the development of a certain kind of relationship between darkness and light. Light certainly illuminates an object in darkness, but darkness also makes light visible. Total darkness, as well as total light, caused by the absence of a contrast between the object and what surrounds it, cannot ever constitute the totality of consciousness, as suggested by the Ganzfeld effect (Schmidt et al., 2020).

From a simplified perspective, we must refer to what has been called object, thing, or something as part of the structure of the world. I will call it *thingness*. Thingness is sub-personal and can be understood from a third-person perspective. Within the framework of the EIT, one can assert that *thingness consists in the materialization of something that extends into space*. The object simply appears when it materializes and extends into space. Although space is crucial for defining the object, the relation is phenomenally unbalanced toward the second component.

I argued that the fundamental property of perceptual consciousness is that its objectual contents extend into space (Forti, 2025). It is what I now refer to as thingness. If an object that extends has a sufficient level of definition, it cannot help but extend in a certain way. Thus, it can be stated that the functional property of consciousness is that its objectual contents extend into the space to which they belong in a certain way. However, to use a comprehensive definition, we must distinguish the property of extending from the property of extending in a certain way. There are properties derived from the former for which the latter is less important.

Consciousness is always consciousness of something (Cleeremans et al., 2025). Franz Brentano (1874/1973) reintroduced the concept of intentionality as the defining feature of mental phenomena: all and only mental acts are intentional, meaning they are directed toward an object that may or may not exist in the real world. It should be noted that, while Brentano’s intentionality is in the third person, subsequent phenomenological developments have inextricably linked it to the subject (Husserl, 1913/1982; Crane, 1998; Jacquette, 2004; Zahavi, 2005; Kriegel, 2016). However, it is not clear what is directed toward an object. What is the mental phenomenon that includes something as an object within itself (Brentano, 1874/1973)? In thingness, the object is contained within a space. It stands in a relation to the surrounding space. In the following sections, we will see how thingness, understood as a relation to space, can also be applied to the subject, and how the mental phenomenon whereby something appears to a subject can be conceived as a construct derived from the relation between objectual contents extended within a space.

Furthermore, we must assume that there is some kind of relation between the mental object and the external object (Twardowski, 1977; Kanizsa, 1979, 1980; Sainsbury, 2017; Bourget and Mendelovici, 2024). If – unlike Brentano – we take an adaptive perspective (Tortoreto, 2022; Crane, 2024), a mental object should possess the characteristics of the knowable object. First of all, it must not possess the characteristics of a face, a flower, or a stone, but of what they have in common, namely, thingness. The object is something to be grasped, or circumvented if it is an obstacle, or avoided if it is dangerous. It is material and extended. The question of whether or not the object exists (Brentano, 1874/1973; Twardowski, 1977; Searle, 1983) must also be understood from this perspective. The imagined world is also part of the knowable world and must possess its fundamental characteristics. We could not create an object without knowing what it is like.

This definition is close to Rubin’s (1915) description of the figure-ground complex. The figure has an object-like character, and there is a tendency to see the figure as positioned in front, and the ground at a further depth plane and continuing to extend behind the figure. Furthermore, the border separating the two segments is perceived as delineating the figure’s shape as its contour, whereas it is irrelevant to the shape of the ground (Todorovic, 2008). What changes is the emphasis on the extended nature of the figure. One could say that in Rubin the extension is implicit, since there can be neither a figure nor a background that is not extended. However, including the extended nature of the figure in the definition means that describing the figure-background relationship becomes a requisite for the occurrence of consciousness.

Secondly, the properties of thingness are similar to the properties traditionally attributed to matter, that is, occupying space and having mass. This view is the opposite of the Cartesian one: from ‘this perspective, consciousness would be a res extensa. This should not be too surprising, since the Cartesian conception of matter – and, to a certain extent, our own – is intuitive and refers primarily to the properties of *perceived* matter. Of course, in the case of consciousness, it is apparent matter. Res extensa is meant in the phenomenological sense of the term. Therefore, we might ask ourselves what the nature of this material appearance is. However, this question is not very different from asking what the ultimate nature of matter is in the world to which we belong (Davies and Gregersen, 2014; Evangelisti, 2023). In this context, the question is relatively important because I am referring to the external reality immediately relevant to an organism’s adaptation. In this sense, the distinction between “point-like” matter, which is dealt with by an NC device, and extended matter, the object of conscious knowledge, is more important due to its functional implications.

In my opinion, thingness, as a fundamental property, has two advantages: (1) it allows for the possibility of proposing physical and biological hypotheses on how it can be realized; and (2) enables a unified view of the main properties of consciousness, as they are derived from it. The other properties relate to the various ways this appearance is realized and how things materialize and extend:

1. Spatial extension is the way in which something material extends into phenomenal space. Consequently, we know how an unknown object is made or how its extension differs from our knowledge of it. In my opinion, this is the most important derived property, because it enables consciousness to acquire a specific functional meaning.
2. A quality is the way in which something materializes based on its degree of differentiation from other qualities. This differentiation depends on their mutual location in a space of characteristics.
3. Thingness is sub-personal because, in order to be conscious, the subject must also be a material entity that extends into space. Furthermore, it must be related to the object in some way. Subjectivity is a structure in which a first-person world and a third-person world, which is formed within the first, overlap and integrate.

In the following paragraphs, I will describe in detail how the three properties of consciousness – spatial extension, subjectivity and feature qualities – can be derived from a fundamental property, and I will explain the functional implications. In fact, the way something appears coincides with knowledge: (1) with spatial extension, we acquire unitary, integrated knowledge of how a content extends; (2) with quality, we know how an extended content materializes, based on its similarity or dissimilarity to another content; (3) with subjectivity, we construct egocentric knowledge that includes the subject itself.

## Spatial extension

Spatial Extension is the property whereby all conscious content extends in some way into the space to which it belongs. This concept has been redefined for the sake of conciseness and to adapt it to the new context. I have described its characteristics in detail in a previous paper (Forti, 2025). Below is a summary of the essential points.

In order to find the simplest form of consciousness, what cannot be eliminated in any way is the object and its relationship to its surroundings (Merleau-Ponty, 1945). In these cases, the usual focus is on the fact that each component of a perceptual field has a quality as an object, background, detail, and so on. But these components share a common property: they are extended. The extended nature of consciousness is more evident in objects. But it is also present in sensations (Husserl, 1913/1982; Merleau-Ponty, 1945), all the more so if we keep in mind that they overlap with objects (Forti, 2024b). Symbols also have a pictorial and extended perceptual nature.

The phenomenal evidence that an object is extended into space carries with it another evidence. If the object is sufficiently defined, identifying its extended nature also means identifying how it extends. To a certain degree of approximation, something similar to what happens in an elementary form of consciousness can be found in the perception of shape on a sheet of paper. It can be useful to ideally refer to an unknown form (Kanizsa, 1979, 1991). When we are visually exposed to such an image, we cannot help but see how the figure occupies the available space of the sheet, in other words how it extends into the space to which it belongs. Remarkably, this ability appears to be independent of the complexity of the form and whether it is known.

Extending in a certain way is not only a property related to how objects appear. As it implies knowledge, it is a property that can be related to conscious function, hence to the handling of information (Lycan, 1996; Mangan, 1998; Earl, 2014). It is possible to propose a more intuitive, albeit rough, definition whereby consciousness enables us to know what the world around us is like, what its structure is. This knowledge needs to be specified both with respect to what it refers (1–2) to and with respect to its characteristics (3–8):

1. By structure I mean the apparent structure of the world, that is, the form, the morphology of the objects we find in it. This means, on the one hand, that it is not an internal or hidden structure. On the other hand, appearance is never limited to a single impression, but is always a structural fact.
2. Form is the way in which an object extends into the space to which it belongs. Extending in a certain way should be understood more broadly, that is, in reference to the image, where not only form in the strict sense, but also the location, size and movement of the object matter.
3. Conscious knowledge is extended knowledge. It is not only knowledge concerning how an object extends. Rather, it is knowledge itself that occurs in an extended form.
4. Conscious knowledge is current knowledge. It is only what we perceive, and therefore know, in conjunction with experience, even if it is influenced by what we already know. “Current” means that knowledge is both almost instantaneous and transient.
5. Knowledge concerns the apparent structure of the world. As such, consciousness has a primarily spatial structure, based on simultaneous acquisition of information. In this, it differs from the structure of a computational system, which is primarily temporal, in that it is based on operations performed sequentially.
6. It is knowledge by images. Forming a conscious image means knowing how an object extends into space. It should be pointed out that conscious images differ from, for example, retinal images or pictorial mental representations. These images are such in that they retain certain topological relations present in the distal stimulus, but they do not in any way guarantee the occurrence of the experience, nor do they have any knowledge by images.
7. Extended knowledge may seem different from form as understood in the common sense of the term. However, the way an object extends into space corresponds to the form as we actually perceive it. We do not define form in this way because our perception of a form is private knowledge. Although we know this, we are not able to specify which way an object extends into space, except to a limited extent (Block, 2011).
8. It is an integrated and unified knowledge. It is a whole made up of interdependent parts. Again, it is an extended integration, different from point-like integration (Tononi, 2004; Tononi and Koch, 2015).

## What is special about subjectivity?

Although I inevitably started from a first-person perspective, in the analysis of the EIT (Forti, 2025) I highlighted a series of structures that can be described in the third person (Box 1). These are third-person structures in the sense that, like the Gestalt laws, they are not based on the relationships with the perceiving subject. However, my approach was limited to vision. Being distal to the subject, vision is more easily objectified. This does not seem possible for more subjective experiences. What is special about subjectivity that does not seem to be present in vision taken in isolation?

It is well known that describing and explaining an experience in the first person is difficult (Chalmers, 1995; Zahavi, 2005; Markic, 2012; Drayson, 2015). According to Nagel (1974), the subjective character of experience cannot be reduced to an objective description. First-person existence is usually considered to be antithetical to third-person existence and exclusive to experience (Searle, 2004). Let us then ask ourselves where this distinction might come from. From the perspective of the EIT, the defining feature of consciousness is that it is a structure whose function is to know the world’s apparent structure.

Let us try to compare consciousness to systems that provide some knowledge of the world, even if that knowledge is far from conscious. For example, a sensor such as a photodiode, a camera, a robot embedded in its own environment, or a self-driving car. Calling them cognitive structures enables us to address the first-person/third-person issue. In these cases, the difference between first- and third-person perspectives is determined by what the knowledge refers to. In the former, it mainly concerns the observing entity and its relationship with the outside world. In the latter, it mainly concerns the observed object because there is still a point of view. An example could be the use of egocentric or allocentric coordinates (Moraresku and Vlcek, 2020).

One could argue that the first-person perspective of experience is something else. That it is something special. In the systems I have listed, there is no distinction between third-person and first-person knowledge, except that one concerns the outside world and the other concerns the observer. However, in the case of consciousness, there seems to be a difference. I have argued that the structure of conscious vision is third-person based on the fact that our visual experience seems to depend almost entirely on the relationships within the visual field. In certain cases, if the point of view is excluded, the subject may be irrelevant and may be set aside (Tononi and Koch, 2008). In fact, this conception of vision does not hold up on its own. There can be no vision, as we experience it, without subjectivity.

Could thingness, understood as the relation of an objectual content to space, also be applied to the subject? If in consciousness knowledge is determined by the relationship between content and surrounding space, let us try to imagine what would happen in a simple conscious structure consisting of a complex of spatial belongings related to a single modality. If there is a single modality – whatever it may be – it is likely that, rather than perceiving how something extends into space, a self-organizing structure *perceives itself* as extending into space in some way. Without an observer, the organism will observe itself. Of course, it would be adaptive for this perspective to concern bodily perception. This experience would be similar to what we all experience when we feel our material body extending into space in some way (Blanke et al., 2015). These types of experiences may be among the first to appear during ontogenetic development in fetuses or newborns (Lagercrantz and Changeaux, 2009).

Conversely, the third-person experience of a visual scenario, which gives us an understanding of the shapes of objects, would be based on the first-person experience of one’s own body. The visual object could appear as such once the visual space overlaps with the peripersonal tactile and proprioceptive space (Noel et al., 2018; Rabellino et al., 2020; Beccherle et al., 2022) and an object is formed within it. It could be argued that in this case the perception of the bodily self would prevail, whereas our experience leads us to turn toward the outside world in most cases. However, the visual object would not behave as a mere satellite of what is happening in the subject. The greater variability of the outside world compared to the bodily world, which occurs in many situations (Legrand, 2007), would shift attention toward the former.

It should be emphasized that, in this description, the primarily subjective nature of conscious perception does not seem to require anything special, but it emerges spontaneously from the very structure of a complex of spatial belongings. One could say that a primary conscious structure, as a structure of knowledge, “is born” in the first person. Instead, it is the third-person perspective that forms from a first-person perspective by superimposing itself as a complex of spatial belongings onto the latter’s space. Of course, we can only speak of first person and subjectivity – even at a rudimentary level – when the perception of the bodily self and the perception of the external world converge (Ronchi et al., 2018).

In an NC cognitive structure, there is no substantial difference between information about the organism and information about the outside world. In consciousness, the difference is constitutive in that a third-person complex of spatial belongings emerges within a first-person complex of spatial belongings. This structural difference corresponds to a significant difference in how we consciously perceive ourselves and the world around us. At the same time, one realizes that object and subject share something in common. For an object to be perceived as such, it must belong to a space. But this also applies to the subject, although in a different way. We cannot perceive our own body unless it belongs to a space, which in this case is the peripersonal space. Both the subject and the object are conscious when they materialize and extend into space. I use the term ‘materialize’ in the phenomenological sense of the term, that is, something that possesses objectual characteristics. This would suggest that the fundamental structure of consciousness is grounded in the relation to the surrounding space, and that thingness is the fundamental property of consciousness.

It should be noted that, although there are points of contact, the distinction between the first and third person differs from the distinction between intentional and intrinsic phenomenology (Henry, 1963; Serban, 2016). The latter refers to the existence or non-existence of something – an object or otherwise – to which experience refers. But in both cases, experience concerns a subject; it is subjective. Thingness, on the other hand, refers to something that can be both the subject and the object and that is primarily in relation to a space. Therefore, it can be conceived in the third person and in objective terms. Subjectivity, as we understand it – that is, the mental phenomenon whereby something appears to a subject – can be conceived as a construct derived from this primary relationship.

## Feature qualities

For simplicity’s sake, I will limit myself to analyzing the feature qualities that manifest themselves as perceptual qualities. Feature qualities are something that materializes in space. In this case as well, the term ‘materialize’ refers to something that possesses objectual characteristics. Even a color, a smell, or a sound are objects when they ‘materialize’ in a space that is not made of colors, smells, and sounds. They represent something that is significant to a living organism and is different from the emptiness of the surrounding space. Extension is also a basic property that sensory qualities must have. If qualities were not extended, they would not be conscious (Merleau-Ponty, 1945). I will analyze the functional meaning of the extended nature of qualities later on.

Furthermore, they overlap with an object or surface (Husserl, 1913/1982). Phenomenal overlapping does not only occur when visual regions overlap with each other. Multisensory stimuli come to us simultaneously from the same region of space. This is reflected in our perceptions, which seem to be formed by overlapping sub-images. A feature quality is something that overlaps with a region of a perceived image – usually an object – characterizing it and modifying the experience (Jerath et al., 2019). Thus, the concept of overlapping allows us to place feature qualities in the context of the relations existing in the field and to assign them a structural role as well as – as we will see – a functional one.

One of the main problems in the approach to consciousness is that we tend to identify the simplest aspects of experience with qualia. It is a common view that simple qualia could be a useful starting point for a theory of consciousness (Koch, 2004). The way in which literature on consciousness has defined the concept of quale over time has coincided with a tendency to separate it from anything having to do with the idea of relationship and structure. Qualia are intrinsic, i.e., non-relational (Dennett, 1988; de Leon, 2001; Siddharth and Menon, 2017).

However, several scholars have examined their relationship with other mental states (de Leon, 2001; Van Gulick, 2017) and, in a broad sense, with the external reality to which they refer (Loar, 2002). From a phenomenological point of view, it is more correct to speak of qualitative aspects as components of experience rather than qualia. What is important is that qualities are related to other components of the experiential field. They cannot be analyzed independently of the experiential field to which they belong. We do not perceive the redness of red as an isolated phenomenon; rather, we perceive something red and thus red as part of the experience. A quality is a quality of something; that is how it presents itself phenomenally. If we consider qualitative aspects taken in isolation as fully representative of experience, and call them “qualia,” we create a phenomenal artifact (de Laguna, 1916).

Relations with other parts of the field can also be conceived as relations with other qualities. These relations have often been defined by the vectors of a space of properties (Churchland, 1989, 1995; Balduzzi and Tononi, 2009). From a phenomenological perspective, conscious experiences form a similarity structure, namely a quality space (Hardin, 1988; Nosofsky, 1992; Clark, 1996; Stanley, 1999; Rosenthal, 2010). The quality space hypothesis about conscious experience proposes that conscious sensory states are experienced in relation to other possible sensory states. For instance, the color red is experienced as being more like orange, and less like green or blue (Fleming and Shea, 2024).

However, questioning the intrinsic nature of qualities and identifying material extension as a fundamental property of any qualitatively connoted content does not allow us to circumvent the hard problem. What cannot be circumvented is the need to explain the qualitative aspects of consciousness. The quality space hypothesis rejects, at least in part, the idea that qualities are something intrinsic. This hypothesis justifies different qualities based on their similarities. However, it does not explain how we perceive a similarity. Unless we adopt the theory of psychoneural identity (Armstrong, 1968; Globus, 1972; Papineau, 2002; Bunge, 2010; Polák and Marvan, 2018), simply placing features in a space is not sufficient to determine a certain quality. There are correspondences between subjective similarity space and neural activation space (Bobadilla-Suarez et al., 2020; Robinson et al., 2023; Moharramipour et al., 2024; Dołega et al., 2025). This does not mean that subjective similarity can be accounted for in terms of neural similarity.

How is similarity realized at the conscious level? An explanation of why we perceive similarities and differences can be found in differentiation. Differentiation is a fundamental process of consciousness. It is necessary for appearance to occur. In order to materialize, conscious content must differentiate itself from the surrounding space. This first form of differentiation occurs at a pre-phenomenal level and does not appear as such (Forti, 2024b). By contrast, the first form of phenomenal differentiation we perceive is the one between figure and background. It is implied in perceptual organization, in which the background is perceived through an external space. In this case, we can truly speak of qualities, which I call Gestalt qualities. They characterize the main object, the background, a detail, or a Gestalt (Forti, 2024b). Gestalt differentiation occurs between the elements that comprise the stimulus field. Although we often overlook this qualitative aspect, it is part of our experience and influences the qualities themselves. For example, the yellow of an object is different from that of the background.

In feature qualities, differentiation occurs between contents with objectual value which do not differ in their role in perceptual organization. Anything that materializes in space differs from anything else that materializes in space, to a greater or lesser extent. It is important to emphasize that quality is affected by various factors (Dołega et al., 2025), from perceptual organization to genetic constraints (Kawakita et al., 2025), from what the individual has learned (Shepard, 1987) to recent memory (Luo and Collins, 2023). When we talk about the quale of red, one relational aspect that is often overlooked is the relationship between the expected stimulus, stored in the individual’s genetic or learned memory, and the actual stimulus (Harris, 2025). Let us bear in mind that qualia imply recognition (Lewis, 1929) and that a new quality tends to be learned and find its place in the feature space (Edelman, 2001).

But in terms of qualities, the comparison is not only with the expected content. It is also with the feature space. Although feature qualities can be identified among elements in the stimulus field, they require activation of the feature space (Lau, 2022). The activation of the feature space creates, at a conscious level, a series of differentiations responsible for perceiving similarities and differences, and ultimately, the qualitative aspects of the experience. On its own, a feature would not possess any quality. A creature that could only ever experience a single flash of light would not experience it as having any particular color (Fleming and Shea, 2024). As more characteristics are arranged in space, a variety of qualities emerge.

It should be emphasized that the nodes that make up the feature space are not equivalent. Some of them represent salient or dominant features and contribute with their relevance to organizing the network in which they are located. According to Fleming and Shea (2024), every aspect of experience has both a relational and a categorical phenomenal character (e.g., color relations as well as intrinsic color). If the match with the expected content is fully achieved, it can be assumed that the differentiation from other nodes in space will be clearer. Consequently, qualities tend to manifest themselves as absolute properties. If the stimulus is close but sufficiently separate, qualities have a more relational character and will be perceived as similar to the reference content. The former probably inspired the concept of qualia, while the latter inspired the idea of qualities being defined by similarity. However, all qualities have a relational character.

## Why and how do physical processes in the brain give rise to feature qualities?

The traditional view of qualia tends to rule out the functional aspect. Many authors do not attribute a function to consciousness (Velmans, 2002; Pockett, 2004; Rosenthal, 2008; Blackmore, 2016; Halligan and Oakley, 2021). However, from an evolutionary perspective, it is difficult to deny its adaptive role (Nichols and Grantham, 2000; Earl, 2014; Kanai et al., 2019; Niikawa, et al. 2022; Ludwig, 2023; Grinde, 2024; Lacalli, 2024; Mogi, 2024; Fitch et al., 2025). The problem of qualia should be closely associated with the—equally unresolved—problem of their functional role (Kanai and Tsuchiya, 2012). Specifically, the function of consciousness should be closely related to the fundamental properties of experience. Otherwise, the risk is to identify a non-conscious function, dissociating the phenomenal aspect from the functional one (Chalmers, 1995; Solms, 2021; Seth and Bayne, 2022).

As my theory is based on collaboration between a conscious and a non-conscious system, with consciousness compensating for certain limitations of NC processes, it is important to understand what the usefulness of the qualities is. Qualities correspond to patterns or features that our organism is able to recognize and that may have adaptive relevance. If we consider their relationship in terms of similarity or difference, they correspond to a point located in feature space. They are not only recognized as corresponding to a pattern, but also differing from other patterns, and they can form classes that encompass them. In other words, they have to do with categorization.

Their conscious functional meaning is hard to identify, at least if we think of simple features that can be handled without problems by an NC processor (Chalmers, 1995; Kanai and Tsuchiya, 2012). The idea of zombies probably arises from a sort of functional equivalence attributed to a presumed epiphenomenal – *and immaterial –* nature of consciousness. Both an NC system and a conscious system seem to have the ability to categorize. They both perform this function, albeit in different ways.

Therefore, the difficulty in attributing a function to the simplest qualia stems from the fact that they appear to be functional duplicates of NC patterns. It remains to be seen whether these duplicates are useful or useless. If we consider that quality implies categorization, then the functional meaning of quality may be that it translates at the conscious level the possibility of knowing *what* is made in a certain way or *what characteristics* it has. In the latter case, we must be aware of the object’s multimodal features, such as its color or taste. It translates because knowledge is already present at the NC level, but it is not connected to the conscious extended knowledge of how the object is made. It is a somewhat accessory function of consciousness, which brings already existing NC knowledge to the conscious level. It is in no way the fundamental function of consciousness, which is to be found in the possibility of perceiving the spatial extension of objects. This may justify the apparent epiphenomenal character of qualia.

However, there is another function closely linked to the first. It directly utilizes the fundamental property of consciousness. Qualitative characteristics are necessarily related to the object. Furthermore, as they are conscious, qualities can only be extended. But they are also extended because they adapt to conscious contents that are extended. This adaptation is useful because it enables us to determine the extent to which their extension corresponds to that of the extended object. A quality does not necessarily affect the entire object uniformly, as is the case with the red color of an apple. In order to relate to the object in relation to their extension, qualities must overlap with it. Their nature as sensations therefore derives, at least in part, from an adaptation to conscious processes involving the possibility of knowing how an object extends. As a result, we will know how a certain quality extends in relation to the extension of the object.

From an evolutionary perspective, consciousness would not initially have developed for the purpose of producing sensations or feelings. Instead, it would have evolved to support more sophisticated sensory systems based on perception, where information is derived from the organization of the stimulus field (Merleau-Ponty, 1945; Kanizsa, 1980). Elementary stimuli, such as those related to certain sensations, would have been included in the conscious perceptual field in order to achieve multimodal completeness of information about the object (Stein and Stanford, 2008). In some cases, the level of detail in the stimulus enables us to determine how a particular color extends across the surface of an object. In other cases, such as with smells or indistinct noises, we only know the approximate extent of the sensation in relation to the extent of the object.

This paper does not aim to investigate the nature of the possible neurobiological mechanisms underlying perceived qualities. Nevertheless, I will propose some preliminary hypotheses to suggest a potential direction for future research. Due to the diversity of the components of the field involved, differentiation manifests itself differently, from pre-phenomenal appearance to Gestalt qualities to feature qualities. However, the nature of differentiation does not change. If the differentiation involved in appearance is not different from that involved in qualities, then the causes of phenomenal qualities must first be sought in what determines appearance itself - in other words, in how something materializes by differentiating itself from the surrounding space. As we have seen with regard to similarity, the factors involved in the various forms of quality are numerous and complex. Consequently, I will limit myself to an explanatory hypothesis concerning pre-phenomenal appearance, that is, the materialization of something that extends into space.

The relationship between an object and its surrounding space cannot be merely topological. I have highlighted the existence of a space that enables us to see, even though the space itself cannot be seen, as well as the existence of content that can be seen (Forti, 2024b). Interactions involving Spatial Belongings are simultaneous, unpredictable, closely integrated and involve the whole field. Therefore it is likely that this process cannot be provided by conventional neuronal organization. Electromagnetic fields could work together with synaptic firing to produce the qualities of early vision (Jones and Hunt, 2023).

It is possible to attribute the nature of forces to these interactions. These forces would also underlie conscious knowledge, that is based on the degree to which an object deviates from a condition of equilibrium in relation to the space to which it belongs (Forti, 2025). One hypothesis of how this might occur is that the system functions as a “weak” force field that produces knowledge rather than changes in the contents of the field.

Therefore, we can assume that the conscious field consists of forces determined by the contrast between concentric regions. As a result, these regions tend to repel each other. Yet even in this case, such forces are unable to change the composition of the elements in the field. Since we are dealing with a relationship between two concentric regions, the outer region behaves differently than the inner region. In the inner regions, the push toward expansion comes from their origin in an inner core. Thus, the existing forces reinforce each other. The external forces, being more widely distributed in space, can only counteract the push toward expansion.

The object tends to expand, but this expansion is limited. It stops at the border of the object. The cognitive approach hypothesizes a process that attributes the ownership of the border to the object (Fang et al., 2009; Chen et al., 2025). However, in a physical conception that searches within the field for the basis of what occurs, the boundary that defines the object must be determined by the space itself. Primary space cannot be seen because it is not apparent. However, the subtraction of visibility (Forti, 2024b) indicates that there is a tendency to make the object visible, which we can attribute to the force of space. If we try to see the background by focusing our attention on it, it is difficult for us to do so, especially near the object, as we are led, somewhat “pushed,” to see the object. This push is consistent with the object’s boundaries.

The inner region pushes outward, tending to expand. At the same time, the outer region pushes inward, limiting its tendency to expand. These opposing pushes could determine the formation of phenomenal matter. Something different happens than in an NC system, which detect a contrast. In consciousness, it is possible not to detect a contrast but *to perceive something* that materializes based on the asymmetric push existing between the opposing forces of two contrasting concentric regions.

This preliminary hypothesis allows for empirical predictions. An increase in contrast between concentric brain regions would lead to an increase in the salience and materialization of content. In turn, an increase in contrast between concentric regions is determined by an increase in contrast within the stimulus field. Similarly, changes in the perception of the content’s extension would be determined by changes in the relationships between the inner and outer regions within the stimulus field.

It should be noted that the scenarios described above could only occur in an NC architecture that makes this possible. For example, these fields could be supported by brain areas whose units are linked by a grid-like connectivity (Haun and Tononi, 2019; Grasso et al., 2021). Furthermore, since Spatial Belongings are organized into a hierarchical structure, the conditions are also determined by the complexity of the underlying architecture. The recently developed Motivated Emotional Mind (MEM) model (Galus, 2026) strongly emphasizes the architecture of representations and neural circuits: (a) hierarchical memory structures encoding knowledge and categories, (b) a fast feedforward sweep as the basis of unconscious recognition and response control, and (c) recurrent processes as the mechanism of phenomenal, reportable consciousness (re-activation of lower sensory fields via feedback). MEM also links consciousness in a keyway to embodiment and motivation: emotions and feelings are understood as forms of perception of internal states, influencing selection and prioritization of contents within the cognitive circuit.

MEM offers an important prerequisite for a mechanism of “materialization”: recurrent activation of lower sensory maps (secondary perception) can “objectify” cognitive content, giving it a perceptual (and thus “thing-like”) character, and “places it” within a spatial structure, rather than leaving it as a purely dispositional representation in higher layers. MEM describes categorization and generalization processes via pattern selection in hierarchical representations, which provides an NC counterpart for a phenomenological description of differentiation. The EIT claims that subjectivity is to arise when modalities (e.g., vision) are superimposed on peripersonal space, creating a third-person perspective within a first-person perspective. An NC counterpart to this organization can be identified in the integration of body and world representations through embodied channels which build a model of the environment and a model of the self as linked structures.

## Concluding remarks

Third-person perspectives are better suited to scientific theorizing (Churchland, 1986; Nagel, 1974; Dennett, 1991; Koch, 2004). On the other hand, consciousness cannot be understood without the first-person perspective (Zahavi, 2005). Several scholars have discussed the possibility of deriving third-person descriptions from a first-person perspective (Varela, 1996; Petitot et al., 1999; Dennett, 2007; Thompson, 2007; Vallor, 2008; Bitbol, 2012; Choifer, 2018; Liou et al., 2024; Jimenez et al., 2025; Northoff and Ventura, 2025; Sandved-Smith et al., 2025). To understand consciousness, we can neither start from third-person data nor simply correlate a first-person event with a biological event in the brain (Sheinberg and Logothetis, 1997; Koch et al., 2016; Polák and Marvan, 2018). Such an event must be conceived, described, and possibly explained in the third person (Drayson, 2015). Moreover, its third-person description must be specific to consciousness itself. Tononi and Koch (2015) state that consciousness has an intrinsic or subjective existence, and provide a third-person description of it. However, this description does not necessarily reflect a subjective experience.

Phenomenology investigates the subjective intentional structure (Husserl, 1913/1982). But if one does not also inquire into what subjectivity consists in—what its structure is—it will always remain an unexplained residue. Starting from visual perception, it has been possible to show that object and subject share something in common: the fact that they must necessarily stand in a relation to a space. Belonging to a space constitutes a structural aspect that underlies subjectivity.

The EIT appears to be able to explain some properties of consciousness, such as subjective bodily extension and feature qualities. Together with the property of extending into space, these properties derive from the fundamental property of consciousness, thingness. These properties highlighted through phenomenal analysis are shown in Box 2 and can be added to those listed in Box 1.

BOX 2 **Additional third-person properties that phenomenal analysis allow us to identify**

- Thingness is the fundamental property of consciousness and consists in the materialization of something that extends into space.

- A mental object must possess the characteristics of the knowable object in order to be directed at it.

- Spatial extension is the way in which something material extends into phenomenal space. This property enables an extended knowledge of images relating to the world.

- Feature quality is the way in which something materializes based on its degree of differentiation from other qualities. It is an accessory function of consciousness and is linked to the extended nature of objectual contents.

- Subjectivity is a structure in which a first-person world and a third-person world, which is formed within the first, overlap and integrate. It allows for an egocentric knowledge that includes the subject itself.

Thingness consists in the materialization of something that extends into space. It derives from the relation between an inner region that has the properties of an object, of a material thing, and an outer region that has the properties of a space in which the object is extended. In this sense, it is subpersonal and it can be understood in the third person. However, it is not opposed to subjectivity. Rather, it constitutes a necessary premise and contributes to explain it. As mentioned, a primary complex of spatial belongings “is born” in the first person. Subjectivity, as a structure in which a third-person world is formed within a first-person world, is a construct derived from something simpler.

The definition of thingness may raise a number of criticisms, not only because it does not include the subject. There are many conscious experiences in which it is difficult to identify anything with a material and extended appearance. These experiences range from scents and emotions to feelings of cold and abstract thoughts. However, I think it is reasonable to assume that the fundamental role of consciousness is to perceive the spatial extension of objects. Qualities, though not objects in the strict sense of the term, nonetheless overlap with objects and surfaces. In its initial evolution, consciousness would be directed primarily towards a material world through a body that is also material. It would be directed towards a world made up of extended objects in order to overcome the limitations of an NC brain directed towards a point-like world.

Its further emotional and cognitive developments would take place in order to act more effectively without ever losing contact with the material world. After all, we cannot experience anything that is not extended. As a rule, emotions are directed towards an object and overlap with it (Arnold, 1960). Symbols, even the simplest ones, are perceived as extended. In order to be adaptive, even an abstract thought must eventually materialize in the world. In any case, in these experiences we continue to perceive, albeit in the background, our body and the world around us. In this respect, consciousness differs from all forms of artificial intelligence with an exclusively symbolic knowledge of the world. The very development of AI, which is much more advanced than robotics, has made it clear that processing without consciousness makes it easier to cope with a point-like world than with an extended world (Moravec, 1988).

The importance of spatial extension has been highlighted by a number of authors. According to Gomez (2025), space is a perceptual construct within consciousness—analogous to the qualia of redness. Haun and Tononi (2019) state that “the subjective feeling of extendedness of the sky or of our own body is just as qualitative and in need of explanation as the feeling of color or pain. It is also pervasive: much of our conscious life is spent anchored in body space, upon which we locate touch or pain, and immersed in visual space, which provides an extended canvas over which all the colors, the contours, and the objects we experience are painted.” However, Spatial Extension is not merely the quale of extension, albeit more important than others. It is the property whereby conscious content extends in some way into the space to which it belongs. Without space, there could be no matter of the object contained within it, nor could the object extend into it.

Subjective perception spontaneously emerges from the processes involved in forming an HSB. In a single modality, a self-organizing structure likely *perceives itself* as extending into space in some way. This initial form of observation would concern the bodily self. This structure interacts with an external object when it is formed in a different modality overlapping with the peripersonal space. Thus, the mental phenomenon whereby something appears to a subject can be conceived as a construct. It is derived from the relationship between objectual contents extended within a space, which I have called Spatial Belongings. The phenomenal structure that manifests itself in perceptual experience is formed by the hierarchical overlapping of Spatial Belongings.

From this perspective, it is not the subject who knows the object. Rather, egocentric knowledge is formed, which includes both the subject and its relationship with the object. The recipient of conscious knowledge is the NC system. According to many theories of consciousness, the functional role of consciousness is such that it can be used by NC systems (Dennett, 1991; Baars, 1997; Crick and Koch, 1998; Dehaene, 2014; Hameroff and Penrose, 2014; Tononi and Koch, 2015; Brown et al., 2019; Seth, 2021; Hunt et al., 2024). Consequently, one might ask why consciousness appears. According to common sense, appearance is how we know the world. If things do not appear to us in order to be known, then why does the property of appearance exist?

Knowledge that is functional to the NC system does not seem to imply the need for appearance. To understand this difference, we must take into account that NC knowledge is based on sensors and algorithms. An NC system can process data, such as distance or intensity gradient, but it must start with something it recognizes. The same applies to measurement, which is only possible if there is a unit of measurement, with its multiples and submultiples, that corresponds to the point being measured. Consciousness “exists” (Tononi and Koch, 2015; Tononi and Boly, 2025) in that it appears as a thing. The property of appearance may be linked to the possibility of knowing new structures without the need to recognize them. In an NC system, something “exists” only when it is recognized. But in consciousness we cannot know how something appears if nothing appears. Therefore, appearance can be a prerequisite for conscious knowledge.

Feature qualities can also be described and understood in the third person. This cannot be applied either to the hedonic and emotional aspects of qualities, or to the aspects that have to do with the bodily self as an agent, which I do not address in this paper. Feature qualities materialize and extend into a phenomenal space, overlapping with objects and surfaces. Qualities are related to other components of the experiential field, as well as to other qualities, forming a similarity structure, or quality space. The hierarchical role of qualities in this space can explain both their relational and categorical phenomenal character (Malach, 2021). The requirements that a feature quality must possess in order to be such are listed in Box 3.

BOX 3 **The requirements that a feature quality must possess in order to be such**

- It must be located as a point-like node in an NC feature space, a multidimensional space.

- It materializes and extends into a phenomenal space, overlapping with objectual contents.

- It is affected by various factors, including the composition of the stimulus field, perceptual organization, genetic factors, and learned ones. One of the necessary factors is the activation of the feature space.

- Differentiation is a fundamental process of consciousness, necessary for appearance to occur. In the phenomenal space, the proximity or distance from other nodes manifests itself as similarity and difference. The feature space becomes a quality space, where a quality materializes in a specific manner, differentiating itself from other qualities.

- Due to their salience, dominant features tend to differentiate themselves from other features in such a way that they are perceived as apparently isolated qualities and therefore as categories. Conversely, when we perceive something that deviates from them, our perception of similarities and differences tends to prevail.

Regarding the hypothesis I have proposed about the possible causes of differentiation, it is a rough preliminary hypothesis that only addresses the pre-phenomenal aspects of consciousness. According to this hypothesis, the primary form of differentiation would be due to the asymmetric action of the forces of two contrasting concentric regions that cannot modify the contents of the field. Phenomenal matter remains a “hard” problem, perhaps slightly less so than asking what underlies the experience of seeing the color red or feeling pain. Even if we answer this question, however, the problem of quality and subjectivity remains in all its aspects. This paper is part of a project that addresses emotions and feelings, as well as the subject as an agent and a motivational entity. I believe it is premature to address the problem of the physical foundations of experience here. First, it is necessary to integrate these latter aspects into the overall framework I have proposed.

## Data Availability

The original contributions presented in the study are included in the article/supplementary material, further inquiries can be directed to the corresponding author.
